# The sorghum *SWEET* gene family: stem sucrose accumulation as revealed through transcriptome profiling

**DOI:** 10.1186/s13068-016-0546-6

**Published:** 2016-06-17

**Authors:** Hiroshi Mizuno, Shigemitsu Kasuga, Hiroyuki Kawahigashi

**Affiliations:** Agrogenomics Research Center, National Institute of Agrobiological Sciences (NIAS), 2-1-2, Kannondai, Tsukuba, Ibaraki 305-8602 Japan; Institute of Crop Science (NICS), National Agriculture and Food Research Organization, 1-2, Owashi, Tsukuba, Ibaraki 305-8602 Japan; Faculty of Agriculture, Shinshu University, 8304 Minami-minowa, Nagano, 399-4598 Japan

**Keywords:** Phloem loading, Phloem unloading, Sugar transporter, Bioethanol, RNA-seq, SNP, Photosynthesis

## Abstract

**Background:**

SWEET is a newly identified family of sugar transporters. Although SWEET transporters have been characterized by using Arabidopsis and rice, very little knowledge of sucrose accumulation in the stem region is available, as these model plants accumulate little sucrose in their stems. To elucidate the expression of key *SWEET* genes involved in sucrose accumulation of sorghum, we performed transcriptome profiling by RNA-seq, categorization using phylogenetic trees, analysis of chromosomal synteny, and comparison of amino acid sequences between SIL-05 (a sweet sorghum) and BTx623 (a grain sorghum).

**Results:**

We identified 23 *SWEET* genes in the sorghum genome. In the leaf, *SbSWEET8*-*1* was highly expressed and was grouped in the same clade as *AtSWEET11* and *AtSWEET12* that play a role in the efflux of photosynthesized sucrose. The key genes in sucrose synthesis (*SPS3*) and that in another step of sugar transport (*SbSUT1* and *SbSUT2*) were also highly expressed, suggesting that sucrose is newly synthesized and actively exported from the leaf. In the stem, *SbSWEET4*-*3* was uniquely highly expressed. *SbSWEET4*-*1*, *SbSWEET4*-*2*, and *SbSWEET4*-*3* were categorized into the same clade, but their tissue specificities were different, suggesting that SbSWEET4-3 is a sugar transporter with specific roles in the stem. We found a putative *SWEET4*-*3* ortholog in the corresponding region of the maize chromosome, but not the rice chromosome, suggesting that *SbSWEET4*-*3* was copied after the branching of sorghum and maize from rice. In the panicle from the heading through to 36 days afterward, *SbSWEET2*-*1* and *SbSWEET7*-*1* were expressed and grouped in the same clade as rice *OsSWEET11/Xa13* that is essential for seed development. *SbSWEET9*-*3* was highly expressed in the panicle only just after heading and was grouped into the same clade as *AtSWEET8*/*RPG1* that is essential for pollen viability. Five of 23 *SWEET* genes had SNPs that caused nonsynonymous amino acid substitutions between SIL-05 and BTx623.

**Conclusions:**

We determined the key *SWEET* genes for technological improvement of sorghum in the production of biofuels: *SbSWEET8*-*1* for efflux of sucrose from the leaf; *SbSWEET4*-*3* for unloading sucrose from the phloem in the stem; *SbSWEET2*-*1* and *SbSWEET7*-*1* for seed development; *SbSWEET9*-*3* for pollen nutrition.

**Electronic supplementary material:**

The online version of this article (doi:10.1186/s13068-016-0546-6) contains supplementary material, which is available to authorized users.

## Background

Sorghum (*Sorghum bicolor*) accumulates sucrose in the stem. This feature is rare among plants, making sorghum a useful source of bioethanol [1–5]. To enhance bioethanol production, it is important to understand and manipulate sucrose phloem loading, unloading, metabolism, and signaling [6–9] and improve the efficiency of bioethanol production [10–13]. Sucrose content generally depends on its metabolism, transport, and storage [14, 15]. The key genes in sucrose metabolism are *sucrose phosphate synthase* (*SPS*) and *sucrose synthase* (*SUS*); their products catalyze rate-limiting steps in this metabolic pathway. The key genes in transport and thus sucrose movement between tissues via the phloem are *sugars will eventually be exported transporters* (*SWEET*) and *sucrose transporters* (*SUT*) [16]. Invertase (INV) is responsible for the degradation of sucrose to glucose and fructose, thus influencing whether sugar molecules are stored as sucrose or starch. These factors synergistically contribute to the stem sucrose content.

SWEET is a newly identified family of sugar transporters [17, 18]. *SWEET* family genes are duplicated, with a diversity of functions: 21–23 *SWEET* genes are known in *S. bicolor*, 17 in *Arabidopsis thaliana*, 18 in *Brachypodium distachyon*, 23 in rice (*Oryza sativa*), 52 in *Glycine max*, and 24 in *Zea mays* [19, 20]. Our knowledge of SWEET has been expanded by using model plants such as Arabidopsis [21] and *O. sativa* [22, 23]. In *Arabidopsis thaliana*, SWEET proteins are located in the plasma membrane or vacuolar membranes and transport sucrose, glucose, fructose, or 2-deoxyglucose; *SWEET* genes are expressed in the leaf, root, flower, seed, and/or pollen [21]. The functions of some of the SWEET proteins have been elucidated: for example, AtSWEET11 and 12 are sucrose transporters responsible for the efflux of photosynthesized sucrose from the leaf, and the double mutant accumulates sugar in the leaf [24]. OsSWEET11, which is essential for reproductive development, are used by the pathogenic bacterium to invade its host [25]. However, because these model plants accumulate little sucrose in their stems, no information on the relationship between SWEET and stem sucrose accumulation is available. Expression of the other sorghum sucrose transporter gene family, *SUT*, differs between Rio (sweet) and BTx623 (grain) sorghum stems [26], but does not differ between Wray (sweet) and Macia (grain) sorghum stems [27]. These findings suggest that *SUT* expression is not a pivotal rate-limiting factor for sucrose transport. To elucidate the mechanism of sucrose accumulation in the stem, it is therefore important to further characterize sorghum *SWEET* family genes.

Our aim here was to characterize sorghum *SWEET* genes using gene expression profiling during the stage of sucrose accumulation. We also used phylogenetic trees to characterize genes and analyzed the synteny of *SWEET* genes between the sorghum and rice chromosomes. We compared the amino acid sequence of SWEET of sorghum SIL-05 (a sweet sorghum used as a material for bioethanol production; [28] and BTx623 (for which a reference genome sequence is available [29], but which is a grain sorghum with lower sucrose content than SIL-05). We also analyzed the expression of other sugar-related genes: *SUT*, *SPS*, *SUS*, and *INV*. We then consider all of the results together to discuss the key genes in phloem loading and unloading and thus accumulation of sucrose in sorghum stems.

## Results and discussion

### Quantification of gene expression during the stage of sucrose accumulation in the stem

**Fig. 1:**
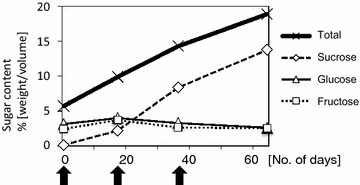
Sugar contents after heading. *Y-axis* indicates sugar content (weight/volume). *X-axis* indicates the number of days after heading, and the *arrow* indicates the point of sampling for RNA-seq

In the sweet sorghum SIL-05, total sugar content increased after heading, reaching 18.9 % on day 64 after heading, whereas the glucose and fructose contents decreased slightly from day 17 (Fig. 1). To identify differentially expressed genes, tissue samples were obtained from the leaf, stem, and panicle on days 1, 17, and 36, respectively, after heading. Their RNAs were then sequenced.

**Fig. 2:**
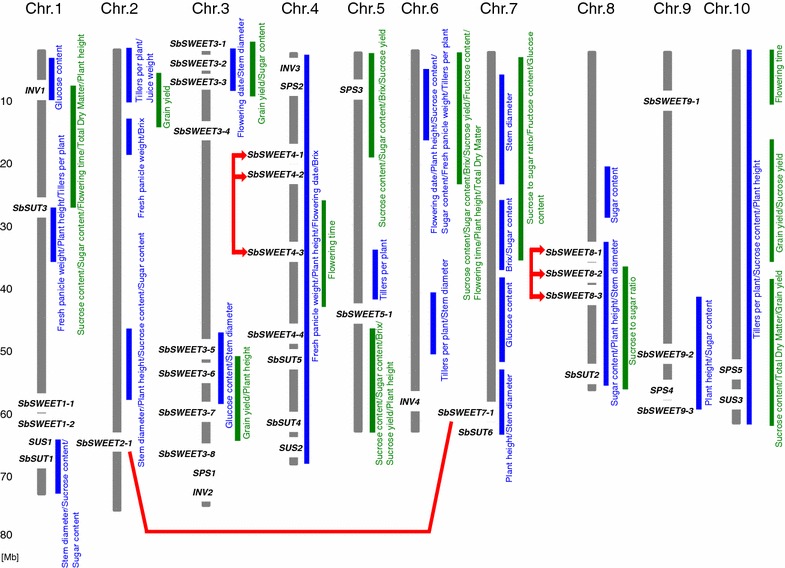
Chromosomal locations of sugar-related genes in sorghum. Chromosomal locations of *SWEET*, *SUT*, *INV*, *SPS*, and *SUS* genes are shown. The positions are based on the reference genome BTx623. Duplicated genes focused in this study are linked by *red lines*. QTLs for sugar-related traits in sweet × grain sorghums are also shown on the basis of two independent QTL analyses: SS79 (sweet sorghum) × M71 (grain sorghum) (*green bars* [30]; and R9188 (sweet sorghum) × R9403463-2-1 (grain sorghum) (*blue bars* [31]). *Numbers* in the column at the *left* indicate the physical lengths of the chromosome [*Mb* megabase]

We focused on genes encoding proteins for sugar transport (SWEET, SUT), sugar metabolism (SPS, SUS), and sucrose degradation (INV) (Table 1). We plotted the chromosomal locations of these genes (Fig. 2) on the basis of their chromosomal locations in the BTx623 genome (Table 1). Quantitative trait loci (QTL) for sugar-related traits that were previously analyzed by using a cross of SS79 (a sweet sorghum) × M71 (a grain sorghum) [30] and R9188 (a sweet sorghum) × R9403463-2-1 (a grain sorghum) [31] are also shown in Fig. 2.

**Table 1:** Sorghum *SWEET* genes, other sugar-related genes, and constitutively expressed genes

Function	Gene name	Gene ID (ver. 2.1)	Gene ID (ver. 1.4)	Chromosomal location
Sugar transporter	*SbSWEET1*-*1*	Sobic.001G373600	Sb01g035490	Chr 1: 58,985,432–58,988,278
	*SbSWEET1*-*2*	Sobic.001G377600	Sb01g035840	Chr 1: 59,380,534–59,384,540
	*SbSWEET2*-*1*	Sobic.002G259300	Sb02g029430	Chr 2: 64,413,792–64,416,541
	*SbSWEET3*-*1*	Sobic.003G015200	Sb03g001520	Chr 3: 1,356,535–1,358,800
	*SbSWEET3*-*2*	Sobic.003G038700	Sb03g003470	Chr 3: 3,617,943–3,620,183
	*SbSWEET3*-*3*	Sobic.003G038800	Sb03g003480	Chr 3: 3,622,464–3,625,366
	*SbSWEET3*-*4*	Sobic.003G149000	Sb03g012930	Chr 3: 15,675,845–15,681,441
	*SbSWEET3*-*5*	Sobic.003G182800	Sb03g024250	Chr 3: 48,309,667–48,324,107
	*SbSWEET3*-*6*	Sobic.003G213000	Sb03g027260	Chr 3: 54,756,647–54,760,169
	*SbSWEET3*-*7*	Sobic.003G269300	Sb03g032190	Chr 3: 60,633,184–60,636,494
	*SbSWEET3*-*8*	Sobic.003G377700	Sb03g041740	Chr 3: 69,215,104–69,218,784
	*SbSWEET4*-*1*	Sobic.004G133500	Sb04g012910	Chr 4: 20,553,590–20,558,352
	*SbSWEET4*-*2*	Sobic.004G133600	Sb04g012920	Chr 4: 20,691,080–20,696,805
	*SbSWEET4*-*3*	Sobic.004G136600	Sb04g015420	Chr 4: 35,162,670–35,166,305
	*SbSWEET4*-*4*	Sobic.004G157100	Sb04g021000	Chr 4: 49,118,793–49,122,120
	*SbSWEET5*-*1*	Sobic.005G123500	Sb05g018110	Chr 5: 44,351,922–44,354,521
	*SbSWEET7*-*1*	Sobic.007G191200	Sb07g026040	Chr 7: 61,176,996–61,180,220
	*SbSWEET8*-*1*	Sobic.008G094000	Sb08g013620	Chr 8: 36,493,752–36,496,643
	*SbSWEET8*-*2*	Sobic.008G094300	Sb08g013840	Chr 8: 36,993,118–36,995,615
	*SbSWEET8*-*3*	Sobic.008G094400	Sb08g014040	Chr 8: 37,249,178–37,251,607
	*SbSWEET9*-*1*	Sobic.009G080900	Sb09g006950	Chr 9: 11,309,919–11,312,702
	*SbSWEET9*-*2*	Sobic.009G143500	Sb09g020860	Chr 9: 50,116,198–50,119,686
	*SbSWEET9*-*3*	Sobic.009G252000	Sb09g030270	Chr 9: 58,680,303–58,682,170
Sugar transporter	*SbSUT3*	Sobic.001G254000	Sb01g022430	Chr 1: 28,168,652–28,172,476
	*SbSUT1*	Sobic.001G488700	Sb01g045720	Chr 1: 68,703,383–68,709,450
	*SbSUT5*	Sobic.004G190500	Sb04g023860	Chr 4: 53,509,428–53,512,882
	*SbSUT4*	Sobic.004G353600	Sb04g038030	Chr 4: 67,476,512–67,481,811
	*SbSUT6*	Sobic.007G214500	Sb07g028120	Chr 7: 63,062,892–63,066,154
	*SbSUT2*	Sobic.008G193300	Sb08g023310	Chr 8: 55,332,646–55,338,922
Sugar degradation	*INV1*	Sobic.001G099700	Sb01g008910	Chr 1: 7,615,347–7,617,621
	*INV2*	Sobic.003G440900	Sb03g047060	Chr 3: 73,993,613–73,997,226
	*INV3*	Sobic.004G004800	Sb04g000620	Chr 4: 439,003–443,225
	*INV4*	Sobic.006G255600	Sb06g031930	Chr 6: 60,211,622–60,214,854
Sugar synthesis	*SPS1*	Sobic.003G403300	Sb03g043900	Chr 3: 71,135,755–71,141,978
	*SPS2*	Sobic.004G068400	Sb04g005720	Chr 4: 5,592,102–5,599,224
	*SPS3*	Sobic.005G089600	Sb05g007310	Chr 5: 12,955,276–12,961,424
	*SPS4*	Sobic.009G233200	Sb09g028570	Chr 9: 57,284,130–57,297,240
	*SPS5*	Sobic.010G205100	Sb10g025240	Chr 10: 54,483,016–54,493,428
Sugar synthesis	*SUS1*	Sobic.001G378300	Sb01g035890	Chr 1: 59,452,295–59,460,141
	*SUS2*	Sobic.004G357600	Sb04g038410	Chr 4: 67,754,722–67,766,746
	*SUS3*	Sobic.010G276700	Sb10g031040	Chr 10: 60,830,697–60,835,335
Control	*actin*	Sobic.008G047000	Sb08g003970	Chr 8: 4,615,047–4,617,619
	*EF1alpha*	Sobic.010G182100	Sb10g023330	Chr 10: 51,879,475–51,882,620
	*GAPDH*	Sobic.010G262500	Sb10g029870	Chr 10: 59,688,771–59,701,308

Chromosomal locations are based on the reference genome Sbicolor_v2.1_255

**Fig. 3:**
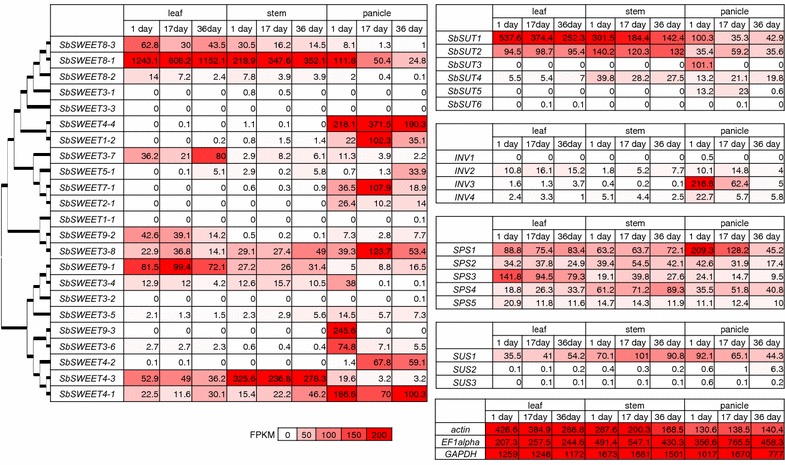
FPKM values of sugar-related genes at the sucrose accumulation stage. FPKM (fragments per kilobase of exon per million mapped sequence reads) values reflect the quantities of existing RNA of each paralog in the cells or tissues. FPKM values for *SWEET*, *SUT*, *INV*, *SPS*, *SUS*, *actin*, *elongation factor 1*-*alpha* (*EF1alpha*), and *glyceraldehyde 3*-*phosphate dehydrogenase* (*GAPDH*) are shown as heatmaps. *Actin*, *EF1alpha*, and *GAPDH* are constitutively expressed controls. The *number* in each *box* indicates the FPKM value of each gene. *Boxes* at the *bottom* indicate the reference color intensities of FPKM values. Samples were extracted from the leaf, stem, or panicle on days 1, 17, and 36 after heading (the stage of sucrose accumulation in the stem). Phylogenetic trees of 23 putative sorghum SWEET genes are also shown on the *left side*

FPKM values were calculated to compare the expression level of each gene. The FPKM of 23 *SWEET* genes, 6 *SUT* genes, 5 *SPS* genes, 3 *SUS* genes, and 4 *INV* genes are shown in Fig. 3. We then focused on the highly and/or differentially expressed genes.

### Identification of SNPs in SIL-05 and BTx623

Single nucleotide polymorphisms (SNPs) were found between SIL-05 and BTx623. Five *SWEET* genes had SNPs that caused nonsynonymous amino acid substitutions between SIL-05 and BTx623 (Table 2). These amino acid substitutions might affect the transport activity of SWEET proteins.

**Table 2:** SNPs and amino acid substitutions in SWEET proteins between the cultivars SIL-05 and BTx623

Gene name	Chromosomal location	Nucleotide		Amino acid	
		BTx623	SIL-05	BTx623	SIL-05
*SbSWEET1*-*2*	Chr 1: 59,381,183	A	T	F	Y
*SbSWEET3*-*7*	Chr 3: 60,636,090	G	A	V	I
*SbSWEET4*-*1*	Chr 4: 20,557,328	G	C	L	V
*SbSWEET4*-*3*	Chr 4: 35,163,171	C	G	E	D
*SbSWEET9*-*3*	Chr 9: 58,680,805	C	G	G	A

### Genes potentially responsible for sucrose accumulation in the stem of sorghum

#### *SbSWEET8*-*1* (*Sobic.008G094000*)

**Fig. 4:**
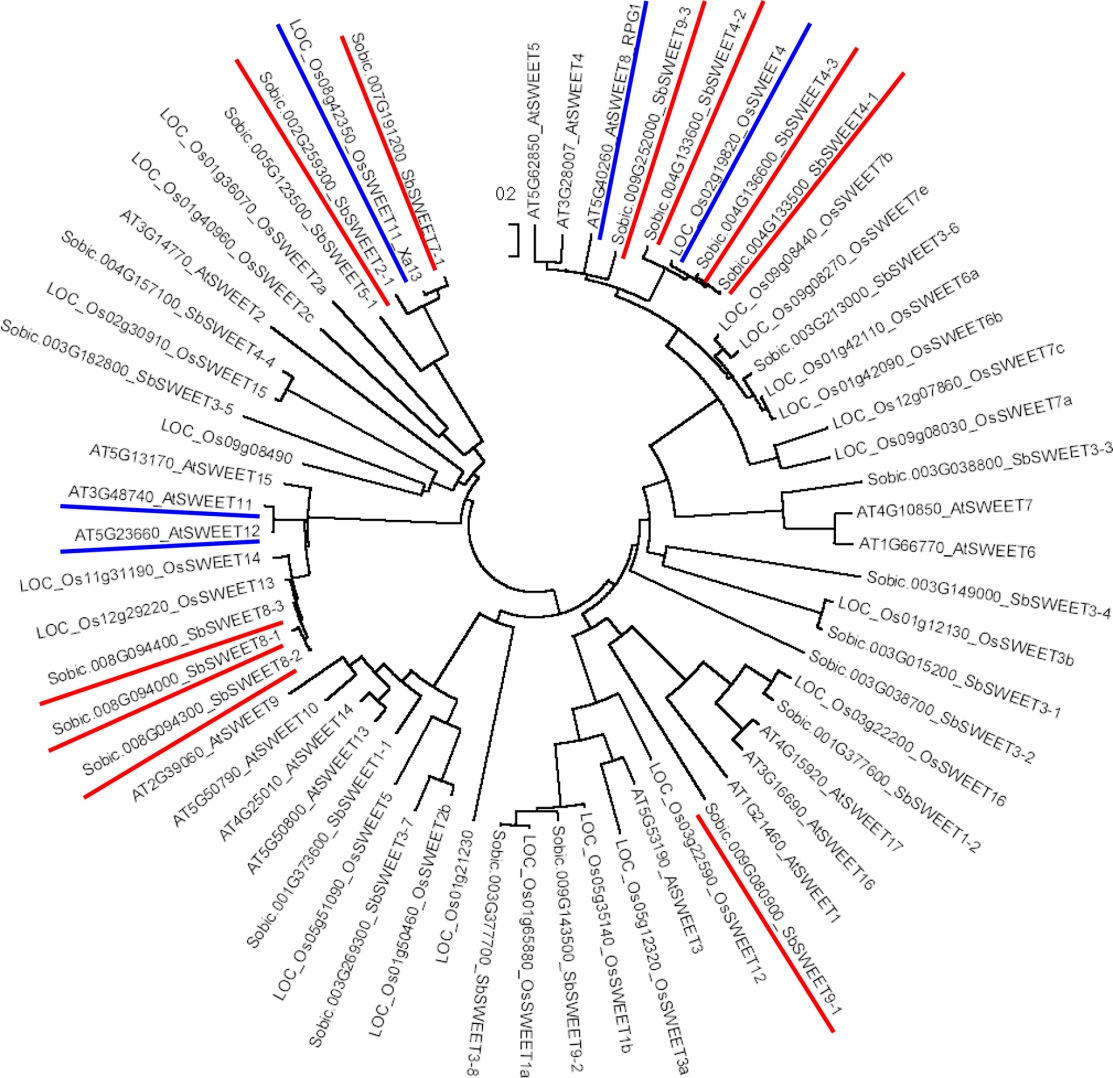
Evolutionary tree of *SWEET.* The tree is drawn to scale, with branch lengths in the same units as those of the evolutionary distances used to infer the phylogenetic tree. This analysis involved 63 amino acid sequences (23 of *Sorghum bicolor*, 17 of *Arabidopsis thaliana*, 23 of *Oryza sativa*). Sorghum *SWEET* genes focused in this study are underlined by *red* and functionally validated Arabidopsis or rice *SWEET* genes are underlined by *blue*

**Fig. 5:**
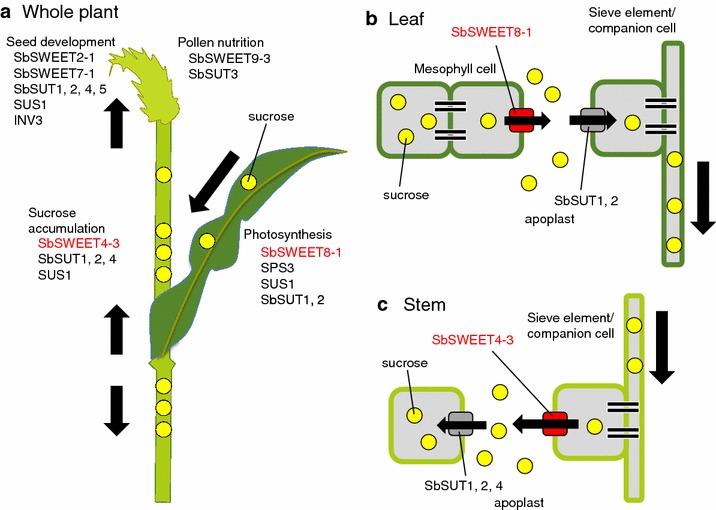
Schematic models of gene expression and roles of SWEET proteins in phloem loading and unloading. **a** Representative genes highly expressed in each tissue during the sucrose accumulation stage; those likely involved in phloem loading of sucrose in the leaf and unloading and accumulation in the stem are shown. **b** Sucrose efflux associated with SWEET proteins in the leaf. Sucrose is synthesized in leaf mesophyll cells and diffuses through the plasmodesmata. SWEET proteins facilitate sucrose efflux into the apoplast. Subsequently, sucrose is taken up and concentrated in the sieve element–companion cell complex by SUT sucrose symporters. Sucrose is transported through the sieve elements out of the leaves to the stem, roots, and seeds. SbSWEET8-1 (Sobic.008G094000) may play a role in the efflux of photosynthesized sucrose to the leaf apoplast. This model was constructed on the basis of an analogy to that in Arabidopsis. **c** SWEET-dependent sucrose accumulation in the stem. Synthesized sucrose is transported from the leaf through the sieve element, and SWEET proteins might facilitate sucrose efflux into the stem apoplast. SbSWEET4-3 (Sobic.004G136600) is a sugar transporter that might contribute to phloem unloading

We examined the genes potentially responsible for sucrose synthesis and efflux from the leaf. *SbSWEET8*-*1* was extremely highly expressed in the leaf from the start of heading through to 36 days after heading (Fig. 3). It was grouped in the same clade as *AtSWEET11* and *AtSWEET12* (Fig. 4). AtSWEET11 and AtSWEET12 play a role in the efflux of photosynthesized sucrose to the apoplast in the leaves of Arabidopsis [21]. Although there were three tandemly duplicated *SWEET* genes (*SbSWEET8*-*1*, *SbSWEET8*-*2*, and *SbSWEET8*-*3*; Fig. 2) in the same clade (Fig. 4), *SbSWEET8*-*1* was the only one expressed at extremely high levels (FPKM > 800 in the leaf; Fig. 3). We therefore consider that it plays a major role in sucrose efflux from the leaf (Fig. 5a, b). Moreover, the gene encoding the enzymes SPS3 (Sobic.005G089600) was highly expressed in the leaf. *SbSUT1* (Sobic.001G488700), a gene encoding an SUT transporter, was highly expressed in the leaf from the start of heading through to 36 days after heading, suggesting that sucrose is taken up and concentrated in the sieve element–companion cell complex (Fig. 5b). These data suggested that sucrose is newly synthesized and actively exported from the leaf at this stage (Fig. 5a).

*SWEET9*-*1* (Sobic.009G080900) was expressed at higher levels in the leaf than in the stem and panicles from the start of heading through to 36 days after heading (Fig. 3). *SWEET9*-*1* had no potential orthologs in Arabidopsis or rice (Fig. 4). Because the expression level of *SWEET8*-*1* was massively higher in the leaf through to 36 days after heading than that of *SWEET9*-*1*, *SWEET8*-*1* may function mainly in phloem loading in the leaf.

#### *SbSWEET4*-*3* (*Sobic.004G136600*)

We next examined the genes potentially responsible for sucrose accumulation in the stem. *SbSWEET4*-*3* was expressed more highly in the stem than in the other tissues during the stage of sucrose accumulation (Fig. 3). Although *SWEET* expression is diverse in the various tissues of sorghum, this potent expression in the stem is unique to *SbSWEET4*-*3* (Fig. 3). *SbSWEET4*-*1*, *SbSWEET4*-*2* and *SbSWEET4*-*3* were located on chromosome 4 (Fig. 2) and categorized into the same clade (Fig. 4). The products of these three *SbSWEET* genes had high levels of amino acid identity with each other (Additional file 1: Fig. S1A), but the tissue specificity was different: *SbSWEET4*-*3* was expressed mainly in the stem, whereas *SbSWEET4*-*1* and *SbSWEET4*-*2* were expressed mainly in the panicles (Fig. 3). We therefore hypothesized that SbSWEET4-3 is a sugar transporter with specific roles in the stem.

We compared the coded sequence and expression of *SbSWEET4*-*3* between SIL-05 and BTx623. One amino acid substitution (D229E) was found between SIL-05 and BTx623 (Table 2). The aspartic acid (D) residue at 229 is conserved as D in the paralogs of SIL-05 (in the case of SbSWEET4-1, SbSWEET4-2, and SbSWEET4-3) (Additional file 1: Fig. S1A), and in other SWEET homologs in *Brachypodium distachyon*, *Oryza sativa*, *Setaria italica*, and *Zea mays* (Additional file 1: Fig. S1B). D229 has also been found in SbSWEET4-3 of other sweet sorghums (Cowley and Top76-6 [32]). We therefore consider that the D residue is necessary for efficient sucrose transport, although some SWEETs might function in tissues other than the stem. Because of the D229E substitution, SbSWEET4-3 in BTx623 might have relatively low sucrose transport activity. Moreover, the relatively high level of expression in the stem of SIL-05 (more than ten times that in the panicle; Fig. 3) differs from that in BTx623: the expression levels of *SbSWEET4*-*3* in the stem of BTx623 at the time of anthesis (150 days after sowing) are as low as those in the panicle [33]. We therefore consider that the amino acid substitution at 229 and the higher level of expression of *SbSWEET4*-*3* in the stem than in the panicle might explain the higher sucrose accumulation in the stem of SIL-05 than in that of BTx623.

**Fig. 6:**
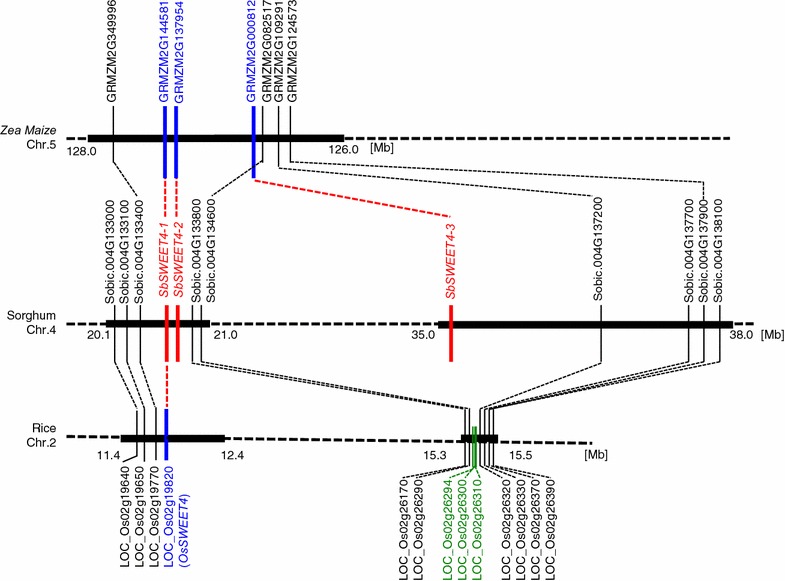
Synteny of *SWEET* genes between chromosome 4 of sorghum, chromosome 5 of maize, and chromosome 2 of rice. Three highly similar sorghum *SWEET* genes were annotated on 20.1–38.0 Mb region of chromosome 4 (*SbSWEET4*-*1,* *SbSWEET4*-*2* and *SbSWEET4*-*3*: in *red*). *Zea maize* has three genes corresponding to *SbSWEET4*-*1,* *SbSWEET4*-*2*, and *SbSWEET4*-*3* on chromosome 5, (*GRMZM2G144581, GRMZM2G137954* and *GRMZM2G000812*, respectively: in *blue*). *SbSWEET4*-*1* corresponds to the rice gene *LOC_Os02g19820* (in *blue*). *SbSWEET4*-*2* and *SbSWEET4*-*3* were likely duplicated from *SbSWEET4*-*1* after the divergence of sorghum and maize from rice. *SbSWEET4*-*3* has no corresponding gene on chromosome 2 of rice. Three rice genes (in *green*) are annotated instead of the *SWEET* homologs. *Numbers* on map indicate locations on the chromosome (*Mb* megabase)

We analyzed the synteny of *SWEET* genes between the sorghum and rice chromosomes using the Plant Genome Duplication Database [34]. The region of chromosome 4 of sorghum had synteny with chromosome 2 of rice, but large insertions/deletions were present, and *SbSWEET4*-*3* had no corresponding *SWEET* genes on chromosome 2 of rice (Fig. 6). In the region corresponding to *SbSWEET4*-*3*, there were three genes instead of putative *SWEET* homologs; the functions of LOC_Os02g26294 and LOC_Os02g26300 are unknown, and LOC_Os02g26310 functions as a leucine-rich repeat receptor-like protein kinase (Fig. 6). We considered that LOC_Os02g19820 (*OsSWEET4*) of rice was the ortholog of *SbSWEET4*-*1*. LOC_Os02g19820 was expressed in leaf, stem, and tissues in the panicle [35]. As the N-terminal region of SbSWEET4-3 was similar to that of SbSWEET4-1 (Additional file 1: Fig.S1A), we thus considered that *SbSWEET4*-*3* was duplicated from *SbSWEET4*-*1* after the branching of sorghum and rice (Fig. 6).

The region also had synteny with chromosome 5 of *Zea mays*. The three sorghum *SWEET* paralogs (*SbSWEET4*-*1*, *SbSWEET4*-*2* and *SbSWEET4*-*3)* corresponded to the three respective maize *SWEET* genes (*GRZM2G144581*, *GRZM2G137954* and *GRMZM2G000812*, respectively; Fig. 6). A comprehensive phylogenetic tree including 777 putative *SWEET* genes of 131 species supports this correspondence [19]. The tissue specificity of the genes was similar to that in sorghum: *GRZM2G144581* is expressed mainly in the embryo; *GRZM2G137954* in the seed and endosperm; *GRMZM2G000812* in the stem 0–30 days after pollination (DAP) [36]. The stem-specific expression after DAP strongly supports the hypothesis that GRMZM2G000812/SbSWEET4-3 has specific roles in the stem. Therefore, gene duplication occurred before the branching of sorghum and maize. Why, then, does maize not accumulate as much sucrose in the stem as sorghum? One hypothesis is that there is a difference in the stem’s capacity to accumulate juice. Some cultivars (such as SIL-05) have a juicy parenchyma in the stem that can effectively accumulate sugar juice, but sorghum cultivars with a dry pith do not [37]. We therefore consider that because maize has a dry pith in the stem, it does not effectively accumulate sugar juice there. We consider that the trait of juicy stem in sorghum is necessary for the accumulation of large amounts of sucrose in this tissue.

We therefore consider that SbSWEET4-3 plays a pivotal role in sweet sorghums because of its potent expression in the stem, the amino acid substitution between SIL-05 and BTx623, and the absence of an orthologous gene in the syntenic region of *O. sativa*. SbSWEET4-3 is a strong candidate for a sucrose transporter that unloads sucrose from the phloem to the stem apoplast during the sucrose accumulation stage (Fig. 5a, c).

#### Other *SWEET genes*

On the basis of their analogy to Arabidopsis or rice genes, we examined *SWEET* genes with functions other than sucrose accumulation.

SbSWEET2-1 (Sobic.002G259300) and SbSWEET7-1 (Sobic.007G191200) were expressed only in the panicle from the start of heading through to 36 days afterward (Fig. 3); these genes are in the same clade as rice *OsSWEET11*/*Xa13* (Fig. 4). *OsSWEET11*/*Xa13* is expressed in the panicle and is essential for reproductive development [23, 25], suggesting that these SbSWEET2-1 and SbSWEET7-1 have roles in seed development (Fig. 5a).

SbSWEET9-3 (Sobic.009G252000) was highly expressed in the panicle only just after heading, after which their expression decreased (Fig. 3). These *SWEET* genes are grouped into the same clade as *AtSWEET8*/*RPG1* (Fig. 4). *AtSWEET8*/*RPG1* is essential for pollen viability through the transport of glucose across the plasma membranes of tapetum cells and pollen cells [21, 38]. We thus consider that SbSWEET9-3 are involved in the transport of glucose and contribute to pollen nutrition in sorghums (Fig. 5a).

#### *SUT*

*SUT* genes are in another sugar transporter family. *SUT* paralogs had tissue-specific expression in sorghum cultivars. In SIL-05, *SbSUT1* and *SbSUT2* were expressed highly in the leaf; *SbSUT1, SbSUT2*, and *SbSUT4* in the stem; and *SbSUT1, SbSUT2,SbSUT3,SbSUT4*, and *SbSUT5* in the panicle. *SbSUT3* was expressed in the panicle just after heading (Fig. 3). In Rio (a sweet sorghum), *SUT2* and *SUT5* are expressed relatively highly in the stem, whereas in BTx623, *SUT5*, and *SUT6* are expressed relatively highly in the inflorescence sink [26]. This tissue-specific expression suggests that *SUT* paralogs function in different sinks—i.e., in either the stem (for sucrose accumulation) or the grain or inflorescence (for starch synthesis or pollen nutrition). However, *SUT* genes are not differentially expressed between Wray (a sweet sorghum) and Macia (a grain sorghum) [27]. Therefore, the high level of expression of *SUT* genes in the stem [26] might be specific to Rio, and not a general feature of sweet sorghums.

#### *INV*

Invertase converts sucrose to glucose and fructose. All four sorghum *INV* genes were hardly expressed, or not expressed in the stem (Fig. 3). Given this absence of INV activity, loaded sucrose would not be hydrolyzed to glucose and fructose, and sucrose would therefore accumulate in the apoplast (Fig. 5). This is consistent with a previous analysis of INV enzymatic activity in the sorghum stem: INV activity in sorghum differs from that in sugarcane, as sugarcane also transfers sucrose to storage parenchyma, with hydrolysis to hexoses by cell-wall INV in the stem [39, 40]. In the panicle just after heading, one sorghum *INV3* (Sobic.004G004800) was highly expressed (Fig. 3); this is consistent with a previous report of the occurrence of cell-wall INV activity in developing seeds [41], suggesting that sorghum INV contributes to starch synthesis in developing seeds.

#### *SUS*

SUS is a sucrose-cleaving enzyme that provides UDP-glucose and fructose [42]. *SUS1* was expressed in all tissues at the sugar accumulation stage (Fig. 3). What is the effect of the sucrose-cleaving enzyme SUS1 in the stem of SIL-05? One hypothesis is that SUS1 provides energy and materials (e.g., cellulose) for construction of the sink structure of the internodes, which in turn increases the sucrose accumulation capacity in the stem of SIL-05. The *SUS* gene in sugarcane (called *SS*) is expressed at high levels in immature (developing) internodes, but at low levels in mature internodes [42]. A second hypothesis is that SUS1 production increases the hexose content of the stem of SIL-05. The hexose (glucose and fructose) content of the stem of SIL-05 (2–3 % each; Fig. 1) is uniquely high among high-Brix sorghums [43]. *SUS1* expression was higher in the stem of SIL-05 (Fig. 3) than in that of BTx623 [33]. The relatively high level of expression of *SUS1* in the stem might therefore relate to the high hexose content of SIL-05.

#### Other genes that might contribute to sucrose accumulation in stems

Expression diversity in sorghum cultivars might be responsible for the characteristic differences in sucrose accumulation between sweet and grain sorghums. Between BTx623 and Keller (a sweet sorghum), 3436 genes are differentially expressed, although 80 % of these differentially expressed genes have orthologs in rice [44]. Sugar-related traits have also been analyzed by quantitative trait locus (QTL) analysis using a cross of SS79 (a sweet sorghum) × M71 (a grain sorghum) [30] or R9188 (a sweet sorghum) × R9403463-2-1 (a grain sorghum) [31]. These traits have been assigned to the ten sorghum chromosomes (Fig. 2), but the genes responsible for the traits have not yet been identified. Here, we showed the chromosomal locations of *SWEET*, *SUT*, *SPS*, *SUS*, and INV (Fig. 2). Some of these genes might be identical to those that were the targets of these previous QTL analyses. However, these genes are not located on the short arm of chromosome 6, even though a QTL analysis [30] indicated that this region was associated with sugar content (Fig. 2). Genes for heading date, plant height, stem diameter, tiller number per plant, panicle weight, and juice weight might also contribute to the final sugar content in sorghum stems. Therefore, genes for these sugar-related agronomic traits will need to be analyzed in the future.

## Conclusions

We determined the expression of key *SWEET* genes for phloem loading and unloading (and thus sucrose accumulation) in sorghum stems. We consider that *SbSWEET8*-*1* plays a key role in the efflux of photosynthesized sucrose from the leaf and that *SbSWEET4*-*3* is a sugar transporter that unloads sucrose from the phloem to the stem apoplast during the sucrose accumulation stage. We also consider that *SbSWEET2*-*1* and *SbSWEET7*-*1* play a key role in seed development and *SbSWEET9*-*3* in pollen nutrition. These *SWEET* genes will be the targets for technological improvement in the production of biofuels.

## Methods

### Plant materials and quantification of stem sugar content

The sorghum cultivar SIL-05 (line number 89) was obtained from Shinshu University in Nagano, Japan. Stem sugar content was measured during the stage at which sucrose is considered to accumulate in the stem (1, 17, 36 and 64 days after heading). The volume/weight of total sugar content, sucrose, fructose, and glucose was measured by capillary electrophoresis and calculated using protocols previously described [43].

### RNA sequencing

RNA was extracted from the second leaf from the flag leaf, the stem (internode only), and the panicle during the stage of sucrose accumulation in the stem (1, 17, and 36 days after heading). Each tissue was immediately frozen in liquid nitrogen and mixed to minimize the effect of transcriptome unevenness among plants. RNA quality was calculated with a Bioanalyzer 2100 algorithm (Agilent Technologies, Palo Alto, CA, USA); high-quality (RNA Integrity Number >8) RNA was used. Sequencing of each 100 bp using an Illumina Hiseq 2000 sequencer (Illumina, San Diego, CA, USA) has been described previously [45, 46].

### Data analysis

Low-quality nucleotides (<Q15) from both the 5′- and the 3′-ends, and adaptors, were trimmed using Cutadapt version 1.0 (https://cutadapt.readthedocs.org/en/stable/). Reads were aligned against sorghum rRNA gene sequences [47] using Bowtie 2 version 2.0.0 beta6 [48]; aligned reads were removed. The remaining reads were aligned to the sorghum reference genome of BTx623 (Sbicolor_v2.1_255) [29] using TopHat version 2.0.4 [49] and Cufflinks version 2.2.0 [50]. FPKM (fragments per kilobase of exon per million mapped sequence reads) values were calculated for each gene model annotated in Phytozome ver.10.3 [51].

### Categorization of the sorghum SWEET gene family

We chose 23 putative sorghum *SWEET* genes in the BTx623 reference genome using the EggNOG database [19]. Even though the number of *SWEET* family genes differs depending on the database (e.g., 21 homologs, [20]; 22 in phytozome 10.3, [51]), the members are nearly consistent. Evolutionary analyses were conducted in MEGA7 [52]. The evolutionary history was inferred using the neighbor-joining method [53]. The evolutionary distances were computed using the Poisson correction method [54] and are in the units of the number of amino acid substitutions per site. All positions containing gaps and missing data were eliminated. The data on chromosomal synteny were based on the Plant Genome Duplication Database [34].

## Additional file

10.1186/s13068-016-0546-6 Amino acid alignment of SWEET proteins. (A) Alignment of SWEET4-1, SWEET4-2, and SWEET4-3 of SIL-05 and BTx623. Red arrows indicate amino acids that differ between SIL-05 and BTx623: V172L of SWEET4-1 and D229E of SWEET4-3. (B) Alignment of putative orthologs of SWEET4-1 among plants. The aspartic acid at 229 of SWEET4-1 (corresponding to position 229 of sorghum SWEET4-3; red arrow) is conserved among putative SWEET orthologs in *Brachypodium distachyon*, *Oryza sativa*, *Setaria italica*, and *Zea mays*.

## Abbreviations

SPS: sucrose phosphate synthase
SUS: sucrose synthase
SWEET: sugars will eventually be exported transporters
SUT: sucrose transporter
INV: invertase
SNP: single nucleotide polymorphism
