# Supplementary material for: The sorghum SWEET gene family: stem sucrose accumulation as revealed through transcriptome profiling
Source: Biotechnol Biofuels. 2016 Jun 17;9:127. doi: 10.1186/s13068-016-0546-6 (PMC4912755; doi:10.1186/s13068-016-0546-6)
Supplement: Supplementary file 1 — 10.1186/s13068-016-0546-6 Amino acid alignment of SWEET proteins. (A) Alignment of SWEET4-1, SWEET4-2, and SWEET4-3 of SIL-05 and BTx623. Red arrows indicate amino acids that differ between SIL-05 and BTx623: V172L of SWEET4-1 and D229E of SWEET4-3. (B) Alignment of putative orthologs of SWEET4-1 among plants. The aspartic acid at 229 of SWEET4-1 (corresponding to position 229 of sorghum SWEET4-3; red arrow) is conserved among putative SWEET orthologs in Brachypodium distachyon, Oryza sativa, Setaria italica, and Zea mays. [file 13068_2016_546_MOESM1_ESM.pdf]

A

```
Sobic.004G133500_SIL05.(SWEET4-1)    1 ----------MVSPDTIRTAIGVIGNGTALVLFLSPVPTFIRIWKKGSVEQYSPIPYVAT    50
Sobic.004G133500_BTx623(SWEET4-1)    1 ----------MVSPDTIRTAIGVIGNGTALVLFLSPVPTFIRIWKKGSVEQYSPIPYVAT    50
Sobic.004G133600_SIL05.(SWEET4-2)    1 MYQSFSTGFFCFVFGAPNPKDVIDGNGTALVLFLSPVPTFVGIWKKRAVEQYSPIPYVAT    60
Sobic.004G133600_BTx623(SWEET4-2)    1 MYQSFSTGFFCFVFGAPNPKDVIDGNGTALVLFLSPVPTFVGIWKKRAVEQYSPIPYVAT    60
Sobic.004G136600_SIL05.(SWEET4-3)    1 ----------MISPDTIRTAIGVIGNGTALVLFLSPVPTFIRIWKKGSVEQYSPIPYVAT    50
Sobic.004G136600_BTx623(SWEET4-3)    1 ----------MISPDTIRTAIGVIGNGTALVLFLSPVPTFIRIWKKGSVEQYSPIPYVAT    50

Sobic.004G133500_SIL05.(SWEET4-1)   51 LLNCMMWVLYGLPVVHPHSMLVITINGTGMAIQLTYVTLFLLYSAGAVRRKVFLLLAAEV   110
Sobic.004G133500_BTx623(SWEET4-1)   51 LLNCMMWVLYGLPVVHPHSMLVITINGTGMAIQLTYVTLFLLYSAGAVRRKVFLLLAAEV   110
Sobic.004G133600_SIL05.(SWEET4-2)   61 LLNCMMWVVYGLPVVHPHSMLVVTINGTGMLIQLSYVVLFILCSTGAVRRKVVLLFAAEV   120
Sobic.004G133600_BTx623(SWEET4-2)   61 LLNCMMWVVYGLPVVHPHSMLVVTINGTGMLIQLSYVVLFILCSTGAVRRKVVLLFAAEV   120
Sobic.004G136600_SIL05.(SWEET4-3)   51 LLNCMMWVLYGLPAVHPHSMLVITINGTGMAIQLTYVTLFLLFSAGAVRRKVVLLLAAEV   110
Sobic.004G136600_BTx623(SWEET4-3)   51 LLNCMMWVLYGLPAVHPHSMLVITINGTGMAIQLTYVTLFLLFSAGAVRRKVVLLLAAEV   110

Sobic.004G133500_SIL05.(SWEET4-1)  111 AFVGAVAALVLTLAHTHERRSMIVGILCVLFGTGMYAAPLSVMKMVIQTKSVEYMPLFLS   170
Sobic.004G133500_BTx623(SWEET4-1)  111 AFLGAVAALVLTLAHTHERRSMIVGILCVLFGTGMYAAPLSVMKMVIQTKSVEYMPLFLS   170
Sobic.004G133600_SIL05.(SWEET4-2)  121 AFVVALAALVLSLAHTHERRSMVVGIVSVFFGTGMYAAPLSVMKMVIETKSVEYMPLFLS   180
Sobic.004G133600_BTx623(SWEET4-2)  121 AFVVALAALVLSLAHTHERRSMVVGIVSVFFGTGMYAAPLSVMKMVIETKSVEYMPLFLS   180
Sobic.004G136600_SIL05.(SWEET4-3)  111 AFVGAVAALVLSLAHTHDRRSMVVGILCVLFGTGMYAAPLSVMKMVIQTKSVEYMPLFLS   170
Sobic.004G136600_BTx623(SWEET4-3)  111 AFVGAVAALVLSLAHTHDRRSMVVGILCVLFGTGMYAAPLSVMKMVIQTKSVEYMPLFLS   170

Sobic.004G133500_SIL05.(SWEET4-1)  171 LASLVNGICWTAYALIRFDLYITIPNGLGVLFAVAQLVLYAMYYKNTQKIIEARKRKTD-   229
Sobic.004G133500_BTx623(SWEET4-1)  171 LASLVNGICWTAYALIRFDLYITIPNGLGVLFAVAQLVLYAMYYKNTQKIIEARKRKTD-   229
Sobic.004G133600_SIL05.(SWEET4-2)  181 LASLANSICWTAYALIRFDVYITIPNGLGVLFALGQLVLYAMFYKNTQQIIEARKRKADH   240
Sobic.004G133600_BTx623(SWEET4-2)  181 LASLANSICWTAYALIRFDVYITIPNGLGVLFALGQLVLYAMFYKNTQQIIEARKRKADH   240
Sobic.004G136600_SIL05.(SWEET4-3)  171 LASLVNGICWTAYALIRFDLYITIPNGLGVLFAVAQLVLYAIYYKSTQEIVEARKRKAD-   229
Sobic.004G136600_BTx623(SWEET4-3)  171 LASLVNGICWTAYALIRFDLYITIPNGLGVLFAVAQLVLYAIYYKSTQEIVEARKRKAE-   229

Sobic.004G133500_SIL05.(SWEET4-1)  230 -QVAMTEVVVDGSGRASNNNTY                                         250
Sobic.004G133500_BTx623(SWEET4-1)  230 -QVAMTEVVVDGSGRASNNNTY                                         250
Sobic.004G133600_SIL05.(SWEET4-2)  241 QQGTVMEVVTDATPPNNNGNTY                                         262
Sobic.004G133600_BTx623(SWEET4-2)  241 QQGTVMEVVTDATPPNNNGNTY                                         262
Sobic.004G136600_SIL05.(SWEET4-3)  230 -QVAMTEVVIDGGKTNNHASGY                                         250
Sobic.004G136600_BTx623(SWEET4-3)  230 -QVAMTEVVIDGGKTNNHASGY                                         250
```

B

```
Sobic.004G133500_SIL05 (SbSWEET4-1)        1 MVSPDTIRTAIGVIGNGTALVLFLSPVPTFIRIWKKGSVEQYSPIPYVATLLNCMMWVLY  60
Brachypodium distachyon_Bradi3g10270      1 MVSPDTIRTAIGVVGNGTALVLFLSPVPTFYRIWKKRSVEQYSAVPYLATLLNCMIWVLY  60
Oryza_sativa_LOC_Os02g19820               1 MVSPDTIRTAIGVVGNGTALVLFLSPVPTFIRIWKKGSVEQYSAVPYVATLLNCMMWVLY  60
Setaria italica_1G135700.1                1 MISPDTIRTAIGVIGNGTALVLFLSPVPTFIRIWKKGSVEQYSPIPYVATLLNCMMWVLY  60
Zea_mays_GRMZM2G000812                    1 MISPDTIRTAIGVIGNGTALVLFLSPVPTFIRIWKKGSVEQYSPIPYVATLLNCMMWVLY  60

Sobic.004G133500_SIL05                   61 GLPVVHPHSMLVITINGTGMAIQLTYVTLFLLYSAGAVRRKVFLLLAAEVAFVGAVAALV 120
Brachypodium distachyon_Bradi3g10270     61 GLPLVHPNSMLVITINGTGMAIELAYVALFLACSAGAARRRVLLILVAEVAFVAAVAALV 120
Oryza_sativa_LOC_Os02g19820              61 GLPAVHPHSMLVITINGTGMAIELTYIALFLAFSLGAVRRRVLLLLAAEVAFVAAVAALV 120
Setaria italica_1G135700.1               61 GLPLVHPHSMLVITINGTGMAIELTYVTLFLLYSTGAARRKVFLLLAAEVAFVGAVAALV 120
Zea_mays_GRMZM2G000812                   61 GLPAVHPHSMLVITINGTGMAIQLTYVALFLLYSVGAARRKVVLLLAAEVGFVGAVAALV 120

Sobic.004G133500_SIL05                  121 LTLAHTHERRSMIVGILCVLFGTGMYAAPLSVMKMVIQTKSVEYMPLFLSLASLVNGICW 180
Brachypodium distachyon_Bradi3g10270    121 LALAHTYERRSMVVGILGVLFGTGMYAAPLSVMKMVIQTKSVEYMPLFLSLASLVNGICW 180
Oryza_sativa_LOC_Os02g19820             121 LNLAHTHERRSMIVGILCVLFGTGMYAAPLSVMKMVIQTKSVEYMPLFLSLASLVNGICW 180
Setaria italica_1G135700.1              121 LSLAHTHERRSMIVGILCVLFGTGMYAAPLSVMKMVIQTKSVEYMPLFLSLASLVNGICW 180
Zea_mays_GRMZM2G000812                  121 LSLAHTHERRSMVVGILCVLFGTGMYAAPLSVMKMVIQTKSVEYMPLFLSLASLVNGICW 180

Sobic.004G133500_SIL05                  181 TAYALIRFDLYITIPNGLGVLFAVAQLVLYAMYYKNTQKIIEARKRK-TDQVAMTEVVVD 239
Brachypodium distachyon_Bradi3g10270    181 TAYALIRFDLYITIPNGLGVMFAVGQVILYAIYYKSTQQILEARKRK-TDQVAMTEVVVD 239
Oryza_sativa_LOC_Os02g19820             181 TAYALIRFDLYITIPNGLGVMFAVAQLILYAIYYKSTQQIIEARKRKEADHVAMTDVVVD 240
Setaria italica_1G135700.1              181 TAYALIKFDLYITIPNGLGVLFAVAQVVLYAIYYKSTQEIIEARKRK-ADQVAMTEVVV- 238
Zea_mays_GRMZM2G000812                  181 TAYALIRFDLYITIPNGLGVLFAVAQLVLYAIYYKSTQEIIEARKRK-ADQIAMTGVVVD 239

Sobic.004G133500_SIL05                  240 GSGRASNNNTY--------  250
Brachypodium distachyon_Bradi3g10270    240 AKNSGAGNGNYL-------  251
Oryza_sativa_LOC_Os02g19820             241 SAKNNPSSGAAAAAANGRY  259
Setaria italica_1G135700.1              239 VGKTNNHAGAGHY------  251
Zea_mays_GRMZM2G000812                  240 GGKTNNQAGAGQY------  252
```
